# CONFIRMing Hepatorenal Syndrome Management: #NephJC Editorial

**DOI:** 10.1016/j.xkme.2021.07.002

**Published:** 2021-08-19

**Authors:** Jamie Willows, Alicja Rydzewska-Rosołowska, Joel M. Topf, Swapnil Hiremath

**Affiliations:** 1 Renal Services, Freeman Hospital, Newcastle Upon Tyne, United Kingdom; 2 Department of Nephrology and Hypertension with Dialysis Unit, Medical University of Białystok, Białystok, Poland; 3 Department of Medicine, Oakland University William Beaumont School of Medicine, Rochester, MI; 4 Department of Medicine, University of Ottawa, Ottawa, ON, Canada

#NephJC is a recurring twitter-based journal club. #NephJC Editorials highlight the discussed article and summarize key points from the NephJC TweetChat.

Traditional journal clubs are fine; one sits with your fellow trainees and attending physicians in the department and one of you presents the article. A few leagues up from this is the nephrology twitter journal club, named “#NephJC.” Here, hundreds of health care professionals, study authors, trainees, content experts, and the occasional patient meet online to share their insights and learn from each other’s experiences in a welcoming environment, straddling specialty silos and flattening medical hierarchy. Geography is no barrier, and attendees join from across the globe and outside academic walls. Ahead of time a team prepares a trial summary as a blog (including a visual abstract), and they host the journal club on twitter, including polls, questions, and a deep-dive discussion of the data. What emerges from this pool representing centuries of clinical experience is the sharing of best evidence and strong opinions in equal measure and the occasional unexpected brilliant perspective that makes everyone rethink their approach to the clinical problem at hand. Having several of the article’s authors contributing is the rule, rather than the exception.^1^

In this installment of the fortnightly #NephJC, physicians with backgrounds in nephrology, hepatology, and intensive care medicine met to discuss the multicenter, randomized, placebo-controlled, double-blinded Confirm Efficacy and Safety of Terlipressin in Subjects With Hepatorenal Type 1 Syndrome (CONFIRM) trial: the largest ever prospective study of patients with type 1 hepatorenal syndrome (HRS-1).^2^ In this commentary, we review the study and some highlights from the twitter discussion.

### The Study

CONFIRM was a phase 3 randomized controlled trial examining the treatment of HRS-1 with terlipressin and albumin versus albumin alone.^2^ HRS-1 is a dreaded complication of end-stage liver cirrhosis and if untreated, it has a median survival rate of only 2 weeks.^3^ The splanchnic vasodilation and decreased systemic vascular resistance that accompany hepatic failure is key in the underlying pathophysiology of HRS-1. Terlipressin is seen as a rational treatment because it is a splanchnic vasoconstrictor, and it has been considered the standard of care for HRS-1 in much of the world for a few years now. However, it does not have approval from the US Food and Drug Administration (FDA) due to risk-benefit concerns highlighted in previous trials, creating the incentive to perform increasingly informative trials examining its efficacy and safety profile in patients with HRS-1. CONFIRM was funded by Mallinckrodt, the drug company holding the licensing rights in the United States, which also designed the trial and performed the data analysis.

CONFIRM was developed with the FDA under a special protocol assessment agreement and was a double-blind placebo-controlled trial. It was conducted over 3 years starting July 2016 at 60 sites in the United States and Canada. Inclusion criteria included the presence of cirrhosis and ascites, serum creatinine value > 2.25 mg/dL, serum creatinine level predicted to double within 2 weeks, and lack of sustained improvement in kidney function 48 hours after diuretic treatment withdrawal and volume expansion with albumin. Patients with sepsis or shock were excluded. Random patient assignment took place in a 2:1 ratio to treatment with terlipressin or placebo, with a deliberately planned higher recruitment to terlipressin treatment to study its safety. Patients would be administered 1 mg of terlipressin as a bolus, or placebo, intravenously every 6 hours, together with albumin (which was “strongly recommended” to be dosed at 1 g/kg of body weight to a maximum of 100 g on day 1 and 20-40 g per day thereafter). Treatment was continued until 24 hours after serum creatinine level became ≤1.5 mg/dL or up to a maximum of 14 days. If serum creatinine level decreased by <30% by day 4, drug dose was increased to 8 mg/d. The primary outcome was “verified reversal of HRS-1,” defined as 2 consecutive serum creatinine measurements ≤ 1.5 mg/dL before day 14 and survival without kidney replacement therapy for an additional 10 days.

A total of 199 patients were randomly assigned to receive terlipressin, and 101 to placebo. Average age was 54 years, mean serum creatinine level was 3.5 mg/dL, and mean serum bilirubin level was 13.1 mg/dL. In the terlipressin group, 32% of patients achieved verified reversal of HRS-1 versus 17% in the placebo group (*P*=0.006). The proportion of patients requiring kidney replacement therapy was also lower in the terlipressin group. However, the number receiving a liver transplant and mortality were no better in the treatment group, and by day 90, death occurred in 51% of the terlipressin group and 45% of the placebo group. An excess of early respiratory failure contributed to poor outcomes in the terlipressin arm, and pooled analysis with previous trials indicated that patients with entry serum creatinine levels ≥ 5 mg/dL were particularly likely to experience harm with terlipressin treatment.^4^

Though the presumption had been that the FDA would approve terlipressin on the basis of reversal of HRS-1, after seeing this additional trial information, they issued a complete response letter indicating that they would not issue a new drug approval for terlipressin and required more information to support a positive risk-benefit profile.^5^ Thus paradoxically, the CONFIRM trial results have further informed views in Europe and other places in which terlipressin is being used for HRS-1, but they also mean that physicians and patients in North America will not have access to terlipressin any time soon.

### The Tweetchat

The NephJC tweetchat took place with 3 editions: American, Asian, and European, and on at least 4 continents, with participation from the nephrology community including some investigators from the trial, as well as some hepatologists and intensivists. The overall numbers report 231 active participants contributing a total of 1,187 tweets. The actual discussion, although structured and moderated, was heated, with many relevant points being raised. Every aspect of the study was analyzed and commented on, including inclusion and exclusion criteria, trial design, primary efficacy end points, and safety concerns. Additionally, many interesting papers were brought up during the chat (eg, about the physiology of head out water immersion^6^).

For eligibility, it was noted that the HRS-1 definition was an older one, which resulted in a "sicker" population with more advanced disease. It was also considered that creatinine-based acute kidney injury definitions would overestimate kidney function in the setting of cirrhosis. The albumin requirement, before eligibility, meant that participants with acute kidney injury due to prerenal factors were correctly excluded; however, the normal-looking serum albumin levels from Table 1 in the study^2^ (∼3.7 to 4 g/dL) were not thought to reflect actual clinical practice. The high doses of albumin used provoked much discussion, with the hindsight bias that increasing preload and afterload simultaneously with a combination of volume and vasopressors might have led to the respiratory adverse events.

During all 3 chats, comments were made on the difficulty of volume assessment and the potential use of point-of-care ultrasound and Venous Excess Ultrasound Score for that particular task.^7^ In the American edition, a lot of attention was paid to mean arterial pressure and its appropriate value as a potential therapeutic target (eg, >85 mm Hg) and the exclusion of those with mean arterial pressures ≤ 70 mm Hg (which was a definition of shock for exclusion). Additionally, though tubular epithelial casts/hematuria was an exclusion criterion, urinary sediment assessment itself is not always performed in routine clinical practice, a point of frequent discussion from a posse of self-declared “pisse prophets” who post interesting urinary sediment images on twitter.^8^^,^^9^

**Figure 1:**
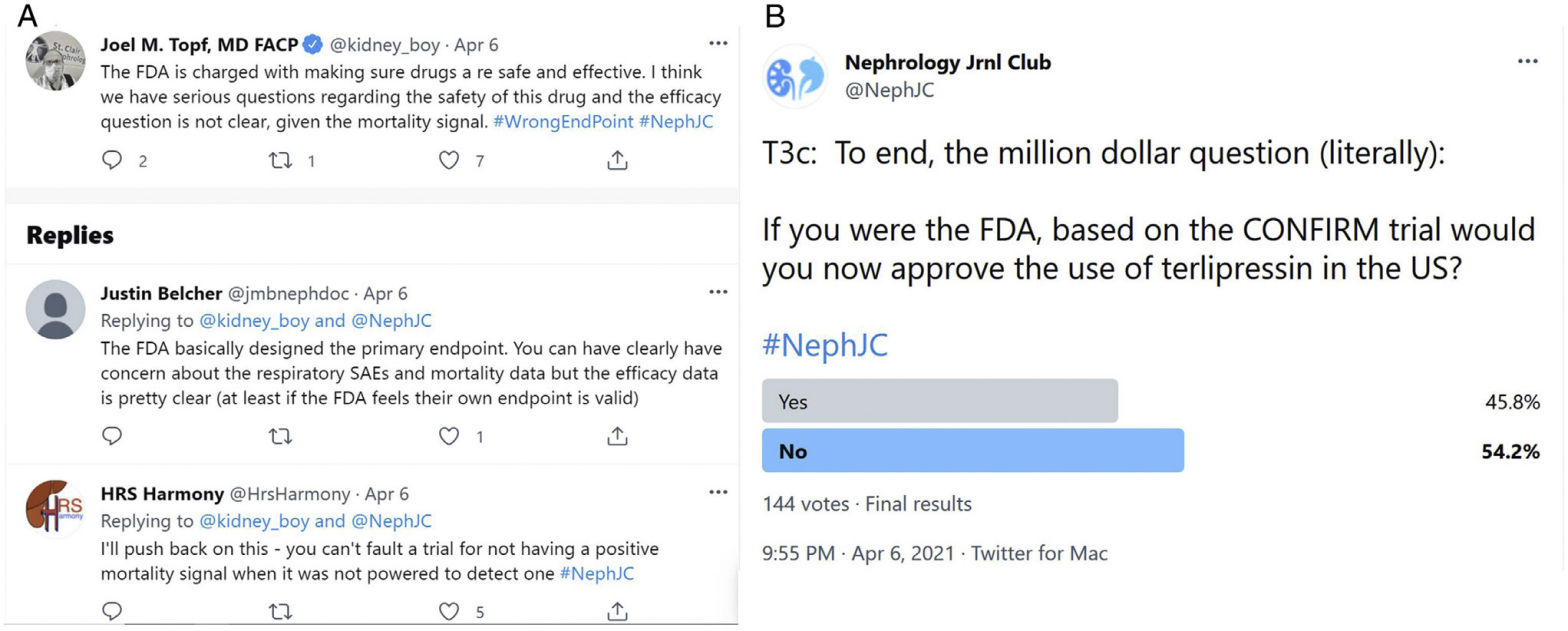
(A) https://twitter.com/kidney_boy/status/1379614322230452226?s=20. Example of a typical twitter conversation, in which Joel Topf questions type 1 hepatorenal syndrome (HRS-1) reversal as an outcome of interest and receives pushback from Justin Belcher that it was designed with input from the US Food and Drug Administration (FDA) and from HRS Harmony that the trial was not powered for a mortality end point. (B) https://twitter.com/NephJC/status/1379613658104401923?s=20. Twitter poll on approval of terlipressin, demonstrating that 54% of 144 respondents agree with the FDA decision. Twitter polls are open to anyone to answer within the period they are open (in this case set at 24 hours). Abbreviations: CONFIRM, Confirm Efficacy and Safety of Terlipressin in Subjects With Hepatorenal Type 1 Syndrome; SAE, serious adverse event.

Though concerns were raised about the use of HRS-1 reversal as the primary outcome and not all-cause mortality, it was quickly pointed out that mortality is rarely the primary outcome in a drug efficacy trial and would require a huge sample size to detect significant mortality differences between groups. In this case, the end point was designed with input from the FDA and has been the commonly used end point in all previous HRS-1 trials (Fig 1). Mortality is measured and reported as a safeguard that a drug does not improve the disease-related primary end point while simultaneously causing increased mortality by another pathway, and in CONFIRM, the measurement and reporting of all-cause mortality thus served its designated purpose.^10^

The American chat was more focused on whether people would like to have terlipressin available. This was driven by arguments that terlipressin use is not restricted to the intensive care unit, this would expand the therapeutic armamentarium for a devastating disease, and HRS-1 reversal is an important outcome by itself because it buys time for liver transplantation to proceed. It was noted that the current standard of care in North America (midodrine and octreotide) is based on a trial of 13 patients, of whom only 5 actually received midodrine and octreotide,^11^ and with the FDA not confirming terlipressin, the possibility of using norepinephrine on the ward was raised, as demonstrated in a recent study.^12^ The Asian and European attendees (with physicians having experience with terlipressin) expressed more concern over the vascular adverse events, and their perception was that respiratory failure was uncommon, possibly because of more judicious albumin use in clinical practice compared with the trial. There was also wide variability in dosing terlipressin for HRS-1 among centers, with some starting at 3 to 4 mg per day and staying at that dose, some going up to 8 mg per day as in the study, and others preferring an infusion rather than a bolus approach. Many specialists in the chat also brought up similar wide variability across the globe in terms of where and by whom the patient is treated (nephrologists, hepatologists, or intensivists) and a different approach to diuretics in HRS-1 among those groups (with hepatologists being more wary of good old furosemide).

Overall, it seems that the enthusiasm for terlipressin use waned after the publication and discussion of the CONFIRM trial. In a twitter poll, most (54%) of the 144 respondents voted to not approve terlipressin for this indication, agreeing with the FDA decision (see Fig 1B).

### Conclusion

Twitter based journal clubs are the go-to place for critical appraisal, especially during a pandemic. In this edition, we were able to discuss the terlipressin data with a multidisciplinary team of experts, including some involved in the actual trial design. The NephJC blog summary also covers not just the main paper, but the paper supplement, manufacturer submission data, and the FDA response letter to make it easy for the interested reader.^4^^,^^13^ Where else could you meet up with people with a passion for nephrology and be able to joke, while discussing cool physiology and the intricacies of trial design? Where else, from the comfort of your own armchair, can you just watch a journal club discussion twist and turn, without having to contribute unless you want to? NephJC is the only institution that lets you experience all the above and more, making learning fun. Remember: twice a month let’s meet up on Twitter to chat, analyze, learn new things, joke, see old friends, and make new ones. Everyone is invited.
